# A New Patient with p40^*phox*^ Deficiency and Chronic Immune Thrombocytopenia

**DOI:** 10.1007/s10875-023-01498-4

**Published:** 2023-05-18

**Authors:** Anna-Lena Neehus, Mathieu Fusaro, NCF4 consortium, Christine Bodemer, Marie Roelens, Adrian Gervais, Jean-Laurent Casanova, Romain Lévy, Jacinta Bustamante

**Affiliations:** 1 Laboratory of Human Genetics of Infectious Diseases, Necker Branch, INSERM U1163, Necker Hospital for Sick Children, Paris, France EU; 2 Paris Cité University, Imagine Institute, Paris, France EU; 3 Study Center for Primary Immunodeficiencies, Necker Hospital for Sick Children, AP-HP, Paris, France EU; 4 Toulouse Institute for Infectious and Inflammatory Diseases (Infinity), INSERM U1291, CNRS U5051, University Toulouse III, Toulouse, France EU; 5 Laboratory of Immunology, CHU Purpan Toulouse, INSERM U1291, CNRS U5051, University Toulouse III, Toulouse, France EU; 6 Pediatric Immunology-Hematology and Rheumatology Unit, Necker Hospital for Sick Children, AP-HP Paris, France EU; 7 St. Giles Laboratory of Human Genetics of Infectious Diseases, Rockefeller Branch, The Rockefeller University, New York, NY USA

To the Editor,

Chronic granulomatous disease (CGD) is an inborn error of immunity (IEI) caused by deficiencies of any of the five NADPH oxidase subunits (p22^*phox*^ (*CYBA*), gp91^*phox*^ (*CYBB*), p47^*phox*^ (*NCF1*), p67^*phox*^ (*NCF2*), and p40^*phox*^ (*NCF4*)) or the chaperone EROS (*CYBC1*), leading to abolished (classic CGD) or abnormal (variant or partial CGD) reactive oxygen species (ROS) production by the patients’ phagocytes [1]. Patients with classic CGD frequently suffer from recurrent, invasive bacterial and fungal infections, whereas patients with autosomal recessive (AR) p40^*phox*^ deficiency have a higher frequency of hyperinflammation and peripheral infections, resembling a milder, variant, atypical form of CGD [1–3].

To date, 26 patients from 14 families with AR p40^*phox*^ deficiency have been described [1, 3]. Most of these patients have inflammatory skin lesions, presenting as lupus-like cutaneous lesions or discoid lupus. About half the patients suffer from inflammatory granulomatous gastrointestinal manifestations, such as inflammatory bowel disease, granulomas, perianal abscesses, and mouth ulcers. Cutaneous infections, mostly due to *Staphylococcus aureus*, were reported in eight of 26 patients, and one patient suffered from disseminated histoplasmosis. The clinical penetrance of p40^*phox*^ deficiency for hyperinflammation or infectious disease is incomplete, at least in childhood, as four of the reported patients remained asymptomatic [1]. Due to the milder clinical phenotype, p40^*phox*^ deficiency is diagnosed later than classic CGD but has a better clinical outcome [1].

We describe here a new case of AR p40^*phox*^ deficiency diagnosed in an 11-year-old girl who had suffered from recurrent mouth ulcers from infancy, chilblain-like purpuric acral lesions, recent photosensitivity, and severe chronic immune thrombocytopenia (ITP). This patient was born to non-consanguineous French parents (Fig. 1a); she was not VZV vaccinated and had experienced an uneventful episode of chicken pox at the age of 4 years. She was vaccinated with the live Bacillus-Calmette Guérin (BCG) vaccine at the age of 5 years and developed local BCG-itis, which resolved spontaneously.

**Fig. 1:**
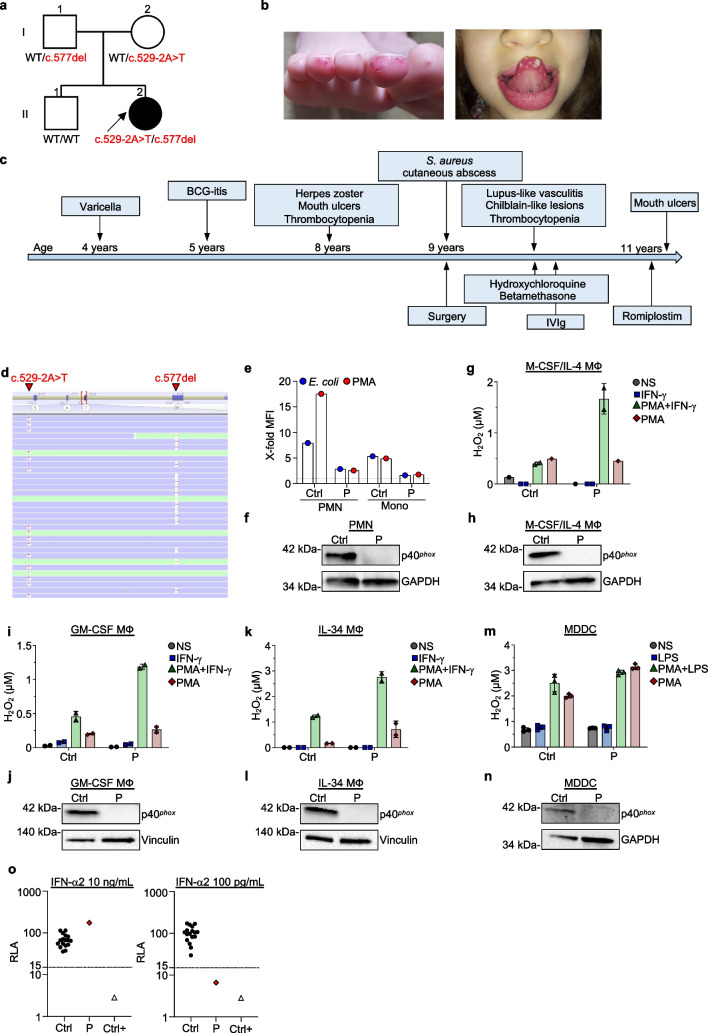
Identification of a new patient with p40^*phox*^ deficiency. **a** Pedigree of the family showing familial segregation of the *NCF4* alleles. **b** Chilblain-like lesions on the patient’s extremities (left) and mouth ulcers (right). **c** Schematic representation of the patient’s clinical history. **d** Alamut viewer presentation of the *NCF4* gene containing the variants for patient **e** quantification of DHR means fluorescence intensity (MFI) of control (Ctrl) and patient (P) neutrophils (PMN) and monocytes (Mono) after stimulation with PMA or *E. coli*, relative to unstimulated control. **f** Western blot for p40^*phox*^ in control (Ctrl) and patient (P) neutrophils (PMN). Extracellular H_2_O_2_ release and p40^*phox*^ expression by **g**, **h** M-CSF-IL-4-derived macrophages (M-CSF/IL-4 MФ); **i**, **j** GM-CSF-derived macrophages (GM-CSF MФ); and **k**, **l** IL-34-derived macrophages (IL-34 MФ) from a healthy control (Ctrl) and the patient (P); technical duplicates ± SD. Extracellular H_2_O_2_ release **(m**) and p40^*phox*^ expression **(n**) by monocyte-derived dendritic cells (MDDC) from a healthy control (Ctrl) and the patient (P); technical triplicates ± SD. **o** Luciferase-based neutralization assay to detect auto-Abs neutralizing 10 ng/mL or 100 pg/mL IFN-α2 using plasma from healthy controls (Ctrl), the p40^*phox*^-deficient patient (P), and an individual known to have neutralizing IFN-α2 auto-Abs (Ctrl +) at a 1:10 dilution. A relative luciferase activity (RLA) < 15% of the value for the mock treatment was considered to correspond to neutralizing activity (dashed line)

At the age of 8 years, the patient presented thoracic monometameric shingles with painless crusty lesions. Whole blood cell counts revealed marked, isolated thrombocytopenia (28 × 10^9^/L), with other blood cell counts within the normal range for the age of the patient (leukocytes: 8800/mm^3^; neutrophils (PMNs): 3000/mm^3^; lymphocytes: 4500/mm^3^; monocytes: 700/mm^3^). The hemoglobin concentration was also normal (11.9 g/dL). Immunophenotyping revealed the proportions of total T, B, and NK cells to be normal, with abnormally high proportions of naïve CD4^+^ T cells and effector memory CD8^+^ T cells and low proportions of central memory and TEMRA CD8^+^ T cells (Table S1). Extensive B-cell immunophenotyping revealed normal proportions of naïve and memory B cells, with no excess of CD21^low^CD38^low^ B cells. Immunoglobulin levels were within the normal range for IgG (12.12 g/L), IgA (1.38 g/L), and IgM (1.52 g/L). Both CRP (2.2 mg/L) and fibrinogen (2.5 g/L) concentrations were normal. The patient tested negative for anti-extractable nuclear antigen, anti-DNA, and anti-Smith autoantibodies. A slightly positive result was obtained for anti-platelet antibodies in the monoclonal antibody-specific immobilization of platelet antigens (MAIPA) assay. A blood smear revealed no morphological abnormalities in the platelets and no schistocytes. The patient also presented recurrent severe mouth ulcers but was otherwise well (Fig. 1b, c). No mucosal bleeding, hematomas, hepatosplenomegaly, or adenopathy was observed.

At the age of 9 years, the patient developed a local thigh abscess due to methicillin-resistant *S. aureus*, which was treated surgically and did not recur. Two months later, she developed lupus-like vasculitis with chilblain-like lesions on her extremities (Fig. 1b, c). The lesions improved rapidly on hydroxychloroquine and betamethasone. Hydroxychloroquine was discontinued after 6 months, and the acral lesions had not recurred at the last follow-up. However, the patient’s thrombocytopenia worsened over time (< 10 × 10^9^/L), with a transient partial (55 × 10^9^/L) response to intravenous immunoglobulin, necessitating second-line weekly subcutaneous romiplostim injections (5 µg/kg/week), which led to a durable complete response. A bone marrow aspiration, performed at the age of 9, showed a polymorphic-rich bone marrow containing megakaryocytes and without cytological abnormalities or signs of hemophagocytosis. Additionally, liver testing for aspartate transaminases (AST) (38 U/L) and alanine transaminases (ALT) (20 U/L) gave normal results, and splenomegaly was ruled out by performing a CT scan at the age of 9. At the last follow-up, the patient was doing well, with no complaints other than a few recurrent mouth ulcers (about two small lesions per month) and mild photosensitivity, and is currently on low-dose romiplostim (3 µg/kg/week), which has made it possible to maintain platelet count at 105 × 10^9^/L. Both her parents and her older brother are healthy.

An IEI was suspected, and the patient was genetically investigated by whole-exome sequencing (WES). Two heterozygous variants were found in intron 6 and exon 7 of *NCF4* in her WES data: c.577del and c.529-2A > T (NM_013416.3), respectively (Fig. 1d). No non-synonymous or copy number variants of any other genes underlying known IEI and compatible with the patient’s clinical phenotype were identified. Familial segregation confirmed an AR mode of inheritance, with the c.577del variant inherited from the patient’s father and the c.529-2A > T from her mother (Fig. 1a). Both variants were predicted to be deleterious, with the first creating a frameshift and a premature stop codon (p.A193Lfs*4) and the second disrupting the splicing acceptor site at the junction of intron 6 and exon 7.

An investigation of the patient’s circulating phagocytes revealed abnormally low levels of ROS production by PMNs and monocytes in response to phorbol 12-myristate 13-acetate (PMA) or opsonized *Escherichia coli* (Fig. 1e). These findings were consistent with previous reports showing that AR p40^*phox*^ deficiency leads to the impairment, but not total abolition, of ROS production in PMNs and monocytes [1–3]. Western blotting of whole-cell lysate of the patient’s PMNs showed an absence of detectable p40^*phox*^ protein, whereas it was readily detectable in healthy control (Fig. 1f). Thus, both *NCF4* variants carried by the patient are deleterious and result in a complete absence of the protein.

We then evaluated the impact of p40^*phox*^ deficiency in monocyte-derived phagocytes. Using isolated CD14^+^ monocytes from the patient and healthy control, we generated three different types of macrophages by priming the cells with GM-CSF (GM-CSF MФ) to induce pro-inflammatory macrophages, or IL-34 (IL-34 MФ) or M-CSF in combination with IL-4 (M-CSF/IL-4 MФ), to favor an M2-like polarization of the macrophages. Consistent with the findings of a previous study, M-CSF/IL-4 MФ generated from the patient’s monocytes displayed normal ROS production (Fig. 1g) [1]. The absence of p40^*phox*^ protein in the patient’s cells was further confirmed by western blotting (Fig. 1h). Interestingly, ROS production was not affected in p40^*phox*^-deficient GM-CSF MФ relative to healthy controls (Fig. 1i, j). Similarly, IL-34 MФ lacking p40^*phox*^ protein displayed normal ROS production after stimulation with PMA in combination with IFN-γ priming (Fig. 1k, l).

We also investigated extracellular ROS production by monocyte-derived dendritic cells (MDDCs) from the patient and found that it was similar to that of control cells after stimulation with PMA (Fig. 1m). The priming of MDDCs with lipopolysaccharide (LPS) for 24 h led to similar levels of ROS production in both patient and control MDDCs, even in the absence of p40^*phox*^ protein expression (Fig. 1n). These results suggest that p40^*phox*^ deficiency does not affect the production of H_2_O_2_ in various subsets of monocyte-derived cells. We did not explore the role of p40^*phox*^ in tissue-resident macrophages or dendritic cells, which could be more relevant for host defense against intracellular pathogens, including mycobacteria [4–6], due to a lack of patient material available from which these cells could be isolated.

The patient presented with herpes zoster at the age of 8 years, which has not been reported previously in patients with p40^*phox*^ deficiency and very rarely in patients with other genetic etiologies conferring CGD. We tested the patient’s plasma for the presence of auto-antibodies (auto-Abs) neutralizing type I IFNs, which were previously shown to underlie viral disease, including herpes zoster [7]. The patient’s plasma contained neutralizing auto-Abs against IFN-α2, neutralizing a dose of 100 pg/mL when used at a 1:10 dilution, which thus could underlie the viral disease observed in this patient (Fig. 1o).

Cutaneous infections and skin inflammation have been observed in more than a third of the reported p40^*phox*^-deficient patients, but this patient is the first to present with chronic ITP [1–3]. ITP is a frequent complication of systemic lupus erythematous and has been observed only rarely in patients with classic CGD or female carriers of heterozygous mutations of *CYBB* [8]. Both these groups are at risk of developing autoimmune manifestations, probably due to their abnormal ROS production in B cells [9]. Patients with classic CGD have been reported to have a low frequency of memory B cells, whereas this patient with atypical CGD displayed no changes in B-cell phenotype during follow-up. Additional studies on B cells from p40^*phox*^-deficient patients and other forms of CGD would help to elucidate the mechanism underlying autoimmunity in these patients.

The clinical penetrance of lupus-like symptoms is incomplete in p40^*phox*^-deficient patients, and additional follow-up studies are required to characterize the clinical spectrum of this disease in more detail, together with the underlying molecular mechanisms. P40^*phox*^ deficiency should be considered in patients with ITP with no clear differential diagnosis to ensure its early detection and appropriate treatment.

## Supplementary Information

Below is the link to the electronic supplementary material.

Supplementary file1 (DOCX 20.5 KB)

## Data Availability

All data and materials can be obtained by contacting the corresponding author.
