# Supplementary material for: A New Patient with p40phox Deficiency and Chronic Immune Thrombocytopenia
Source: J Clin Immunol. 2023 May 18;43(6):1173–7. doi: 10.1007/s10875-023-01498-4 (PMC10354143; doi:10.1007/s10875-023-01498-4)
Supplement: Supplementary file 1 — Supplementary file1 (DOCX 20.5 KB) [file 10875_2023_1498_MOESM1_ESM.docx]

**Supplementary information**

**Table S1. Immunophenotyping of the p40*^phox^*-deficient patient**

| Subset | | Relative (%) | | Cells/µL | | |
| --- | --- | --- | --- | --- | --- | --- |
|  |  | Patient | Reference | Patient | | Reference |
|  |  | Age 8 |  | Age 8 | |  |
| T cells | CD3^+^ | 62 | 60-76 | 2310 | | 1200-2600 |
|  | CD4^+^ | 34 | 31-47 | 1266 | | 650-1500 |
|  | CD8^+^ | 24 | 18-35 | 894 | | 370-1100 |
|  | CD45RO^+^/CD4^+^ | 21 | 13-30 |  | | |
|  | CD45RA^+^/CD4^+^ | 79 | 58-70 |  |  |  |
|  | CD31^+^/CD4^+^ | 61 | ND |  |  |  |
|  | CD31^+^CD45RA^+^/CD4^+^ | 60 | 43-55 |  |  |  |
|  | CCR7^+^CD45RA^+^/CD8^+^ | 63 | 52-68 |  | | |
|  | CCR7^+^CD45RA^-^/CD8^+^ | 2 | 3-4 |  |  |  |
|  | CCR7^-^CD45RA^-^/CD8^+^ | 29.5 | 11-20 |  |  |  |
|  | CCR7-CD45RA^+^/CD8^+^ | 5.5 | 16-28 |  |  |  |
| B cells | CD19^+^ | 19 | 4.8-24.3 | 708 | 219-509 | |
|  | CD27^+^/CD19^+^ | 8 | 9-35 |  | | |
|  | CD27^-^IgD^+^/CD19^+^ | 70 | 58.5-84.6 |  |  |  |
|  | CD27^+^IgD^+^/CD19^+^ | 4 | 3-21.1 |  |  |  |
|  | CD27^+^IgD^-^/CD19^+^ | 4 | 4.4-20.5 |  |  |  |
|  | CD21^++^CD24^+^/CD19^+^ | 71 | ND |  |  |  |
|  | CD24^++^CD38^++^/CD19^+^ | 13 | 4.5-9.2 |  |  |  |
|  | CD24^++^CD38^++^CD27^-^IgD^+^/CD19^+^ | 13 | 13-36 |  |  |  |
|  | CD24^-^CD38^++^/CD19^+^ | 0.4 | 5 |  |  |  |
|  | CD21^-^CD38^-^/CD19^+^ | 3 | 4 |  |  |  |
| NK cells | CD16^+^CD56^+^ | 18 | 4-17 | 670 | | 100-480 |

ND: Not determined.

**Material and Methods**

**Differentiation of monocyte-derived cells**

CD14^+^ cells were isolated from peripheral blood mononuclear cells using anti-CD14 Microbeads (Miltenyi Biotec) according to the manufacturer’s instructions. Differentiation into monocyte-derived dendritic cells was achieved by culture of isolated CD14^+^ cells in RMPI 1640 supplemented with 10% FCS, IL-13 (10 ng/mL; 213-ILB, R&D Systems) and GM-CSF (50 ng/mL; 215-GM, R&D Systems) for 7 days. For GM-CSF-derived monocyte-derived macrophages (Mφ), CD14^+^ cells were cultivated in M1-Macrophage Generation Medium XF (PromoCell) for 7 days according to the manufacturer’s instructions. M-CSF/IL-4 Mφ differentiation was induced by culture in RPMI 1640 supplemented with 10% FCS and M-CSF (50 ng/mL; #216-MC, R&D Systems) for 7 days, followed by 7 days of M-CSF and IL-4 (50 ng/mL; #204-IL, R&D Systems) treatment. Differentiation with IL-34 (50 ng/mL; #200-34, Peprotech) was performed in RPMI 1640 supplemented with 10% FCS for 7 days.

**ROS production assays**

ROS production by neutrophils and monocytes was analyzed by incubating whole blood with dihydrorhodamine 123 in the presence or absence of PMA or *E. coli* at 37°C for 20 minutes. Red blood cells were lysed with ammonium chloride lysis buffer (80.2 g NH_4_Cl, 8.4 g NaHCO_3_, 3.7 g EDTA). Analysis was performed on a Gallios flow cytomter (Beckman Coulter). Extracellular production of H_2_O_2_ by monocyte-derived cells was assessed with or without PMA stimulation (400 ng/mL) using the Amplex Red Kit (#A22188, Thermo Fisher Scientific) in Krebs-Ringer bicarbonate buffer (#K40002, Merck). Briefly, 3x10^4^ cells were seeded in 96-well flat bottom plates and primed with 1000 IU/mL human recombinant IFN-γ (Imukin, Boehringer Ingelheim) or 1 µg/mL LPS (*Salmonella minnesota*; #L6261, Merck) for 16 hours before the experiment. H_2_O_2_ release was quantified with a Victor Nivo plate reader (Perkin Elmer).

**Protein isolation and Western blotting**

Neutrophils were isolated after ficoll density gradient centrifugation with subsequent red blood cells lysis using ammonium chloride lysis buffer. Whole cell lysates of neutrophils and monocyte-derived cells were prepared with modified radioimmunoprecipitation assay buffer (25 mM Tris-HCl pH 7.4, 150 mM NaCl, 1% NP-40 and 1 mM EDTA) supplemented with protease inhibitor cocktail (#5892970001, Merck), 0.1 mM DTT (#20290, Thermo Fisher Scientific), 1 mM PMSF (#10837091001, Merck) and phosphatase inhibitor cocktail (#4906837001, Merck). Protein concentrations were determined by Bradford protein assay, and 30 µg of protein were subjected to SDS-PAGE using 10% Criterion TGX pre-cast gels (#5671034, BIO-RAD). Proteins were transferred onto nitrocellulose membranes (#1704159, BIO-RAD) with the Trans-Blot Turbo Transfer System (#1704150, BIO-RAD), followed by bocking with 3% BSA in PBS-T. Membranes were then incubated with antibodies directed against p40*^phox^* (1:1000; #07-503, Merck), GAPDH (1:3000; #sc-47724, Santa Cruz Biotechnology) or vinculin (1:3000; #sc-25336, Santa Cruz Biotechnology) and visualized with the Clarity Western ECL substrate (#1705061, BIO-RAD) or SuperSignal West Femto (#34096, ThermoFisher Scientific) with the ChemiDoc MP (BIO-RAD). Images were analyzed with Imagine Lab 5.1 (BIO-RAD).

**Luciferase reporter assays**

The blocking activity of anti-IFN-α2 auto-Abs was determined with a reporter luciferase activity. Briefly, HEK293T cells were transfected with a plasmid containing the Firefly luciferase gene under the control of the human ISRE promoter in the pGL4.45 backbone, and a plasmid constitutively expressing Renilla luciferase for normalization (pRL-SV40). Cells were transfected in the presence of the X-tremeGene9 transfection reagent (#6365779001, Sigma-Aldrich) for 24 hours. Cells in Dulbecco’s modified Eagle medium (DMEM, Thermo Fisher Scientific) supplemented with 2% fetal calf serum (FCS) and 10% healthy control or patient serum/plasma (after inactivation at 56°C, for 20 minutes) were either left unstimulated or were stimulated with IFN-α2 (#130-108-984, Milteny Biotec) at 10 ng/mL or 100 pg/mL for 16 hours at 37°C. Finally, cells were lysed for 20 minutes at room temperature and luciferase levels were measured with the Dual-Luciferase® Reporter 1000 assay system (#E1980 Promega), according to the manufacturer’s protocol. Luminescence intensity was measured with a VICTOR-X Multilabel Plate Reader (PerkinElmer Life Sciences, USA). Firefly luciferase activity values were normalized against Renilla luciferase activity values. These values were then normalized against the median induction level for non-neutralizing samples, and expressed as a percentage. Samples were considered neutralizing if luciferase induction, normalized against Renilla luciferase activity, was below 15% of the median values for controls tested the same day.
